# Robotic-assisted laparoscopic repair of a symptomatic uterine isthmocele following cesarean delivery: a case report

**DOI:** 10.1093/jscr/rjag288

**Published:** 2026-04-22

**Authors:** Mishalle Rashid, Daniel Rashid, Ahan L Hunter

**Affiliations:** Edward Via College of Osteopathic Medicine, Virginia Campus, 2265 Kraft Drive, Blacksburg, VA 24060, United States; George Mason University, 4400 University Drive, Fairfax, VA 22030, United States; Department of Obstetrics and Gynecology, About Women OB/GYN / Sentara Northern Virginia Medical Center, 2300 Opitz Boulevard, Woodbridge, VA 22191, United States

**Keywords:** isthmocele, cesarean scar defect, robotic surgery, laparoscopic repair, pelvic pain

## Abstract

A uterine isthmocele, or cesarean scar defect, is a complication of cesarean delivery. This report presents a 36-year-old female patient with chronic pelvic pain and uterine bleeding years after a cesarean delivery. Initially, conservative treatment did not alleviate these symptoms. Upon failing conservative measures for symptom relief, she elected to undergo a definitive surgical repair. The patient underwent diagnostic hysteroscopy followed by robotic-assisted laparoscopic repair. Intraoperative evaluation revealed a 2–3 cm niche at the lower uterine segment, which was excised and reconstructed in two layers. Bilateral tubal patency and a watertight closure were confirmed with methylene blue instillation. The procedure was completed without complications and with minimal blood loss. Postoperative recovery was uncomplicated, and the patient reported early symptom improvement. This case demonstrates a minimally invasive surgical technique in a patient with a symptomatic isthmocele and serves to inform osteopathic practitioners of considerations when encountering patients with post-cesarean sequelae.

## Introduction

Uterine isthmocele is a defect in the myometrium at the site of a previous cesarean incision and has been documented in 24%–70% of patients depending on imaging modality and diagnostic criteria, based on studies evaluating several hundred post-cesarean patients [1]. Although frequently asymptomatic, clinically significant defects may lead way to abnormal uterine bleeding, chronic pelvic pain, or secondary infertility [2]. The underlying mechanism is thought to involve inadequate myometrial healing and the development of a pouch-like indentation that retains menstrual blood [3].

Single-layer uterine closure, retroflexed uterine position, multiple cesarean deliveries, low transverse incisions, and other variations in surgical technique are associated risk factors [4, 5]. Double-layer closure has been associated with improved myometrial approximation and reduced niche formation [6]. Transvaginal ultrasonography and saline infusion sonohysterography are the most frequently used methods in order to evaluate the depth, width, and thickness of residual myometrium in the niche [7, 8]. Lower residual myometrial thickness, often cited at <3 mm, is often associated with higher symptom intensity [9].

Management options include hormone or surgical treatment. Hysteroscopic repair can be considered in smaller defects with existing uterine wall thickness, but defects in deeper regions with a thin (<3 mm) residual myometrial thickness (RMT) may need laparoscopic or robotic repair [10, 11]. Despite multiple operative approaches described in the literature, the optimal surgical technique for symptomatic isthmocele remains debated, particularly in patients with significant myometrial thinning. Additionally, robotic repair offers better vision, dexterity, and accuracy for complete removal and repair [12, 13].

This case report describes a patient with a symptomatic isthmocele refractory to conservative management and highlights the role of robotic-assisted laparoscopic repair as a minimally invasive option in this clinical setting.

## Case report

The patient was a 36-year-old White woman presenting with chronic pelvic pain and abnormal uterine bleeding. Her menstrual cycles came every 28 days, with bleeding lasting 4–5 days followed by spotting and cramping. She also detailed intermittent right-sided pelvic pain. Symptoms gradually worsened despite the use of combined oral contraceptives.

### History and examination

**Figure 1:**
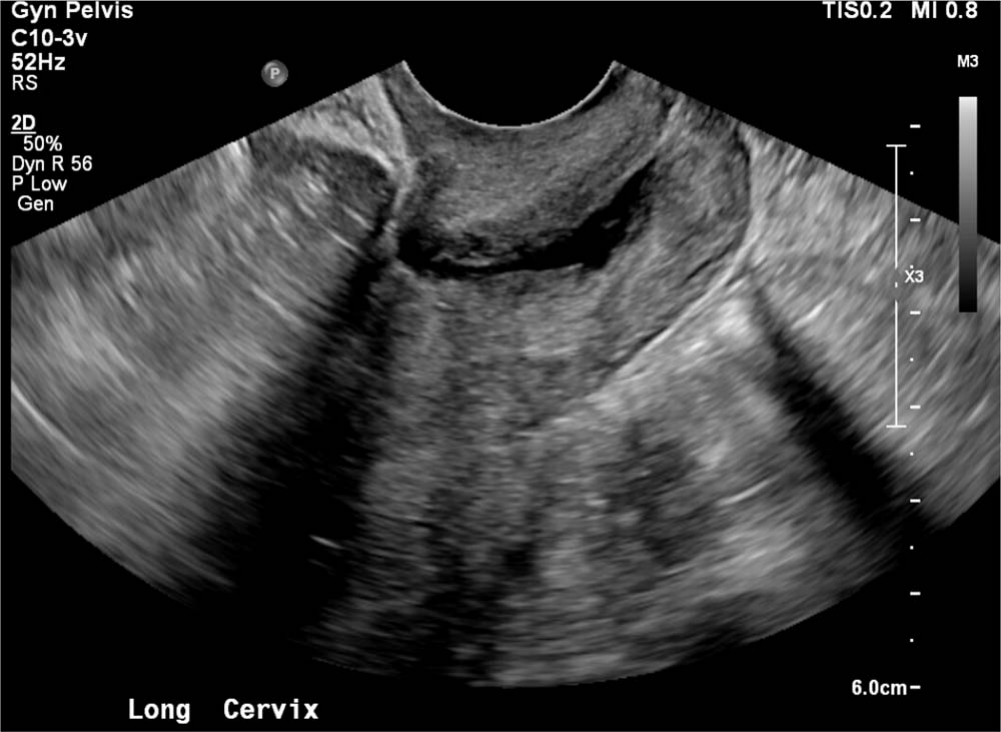
Transvaginal ultrasound image of uterine cavity prior to isthmocele identification during laproscopic procedure.

Past medical history included anxiety, major depressive disorder, insomnia, asthma, right ovarian cyst, and chronic pelvic pain. Surgical history included one low transverse cesarean delivery in 2020 and a benign cystoscopy in 2021. She was G1P1, sexually active, and used oral contraceptives. She smoked daily and denied alcohol or illicit drug use. Vital signs were normal. The abdomen was soft and non-tender. Pelvic examination findings were unremarkable in prior evaluations. Transvaginal US was unremarkable (Fig. 1).

### Diagnostic evaluation

Evaluation suggested a symptomatic lower uterine segment defect consistent with isthmocele. Definitive assessment was planned through hysteroscopic and laparoscopic visualization.

### Surgical procedure

On 18 March 2025, the patient underwent diagnostic hysteroscopy followed by robotic-assisted laparoscopic repair under general anesthesia. The hysteroscopy showed a defect in the lower uterine segment typical of a niche. Robotic laparoscopy was then performed with infraumbilical Veress entry and 8 mm trocar placement.

Pelvic survey revealed normal adnexa aside from the defect. A bladder flap was created, exposing a 2–3 cm indentation at the prior hysterotomy site. The defect was excised using cold scissors and electrocautery, and the specimen was removed in an EndoCatch bag.

The uterine wall was layered using two layers of 2-0 V-Loc barbed sutures. Transcervically, methylene blue dye was injected, which demonstrated the absence of leakage and bilateral tubal patency. Port sites were closed with 4-0 Monocryl. Estimated blood loss was 15 mL. No complications occurred.

### Postoperative course

Recovery was uncomplicated. No postoperative imaging or additional diagnostic testing was performed following surgical repair. The patient was advised to avoid intercourse, strenuous activity, and heavy lifting for 2 weeks. She reported improved pelvic discomfort and reduced abnormal bleeding at 2-week postoperative follow-up.

## Discussion

In the context of this patient’s presentation and management course, previously described characteristics of uterine isthmocele help frame the rationale for operative intervention. Uterine isthmocele is a postoperative complication of cesarean delivery associated with abnormal uterine bleeding, chronic pelvic pain, and infertility [14]. The pathophysiology is related to impaired myometrial healing that causes a pouch-like indentation in the anterior wall of the uterus.

Single-layer closure, retroflexed uterine position, and multiple prior cesarean deliveries are risk factors that enhance the likelihood of niche formation. Transvaginal ultrasonography is most often employed in diagnostic evaluation, with adjunct techniques used to further characterize niche morphology [7, 8, 15].

Operative management may be necessary in symptomatic patients. Small, shallow defects are amenable to hysteroscopic resection, but deeper defects or those with thin myometrium will require laparoscopic or robotic reconstruction. Robotic-assisted repair offers improved visualization and allows for precise complete excision of the defect with a layered closure.

Uterine isthmoceles have significant implications for future reproductive planning and obstetric risk beyond symptom burden, which represents further rationale for accurate diagnosis and appropriate management [1]. Defects involving considerable myometrial atrophy have also been linked with cesarean scar ectopic pregnancy, uterine rupture in subsequent gestations, and improper placentation, including disorders of the placenta accreta spectrum [4].

These are factors show the utility of restoring myometrial status for those desiring future fertility or experiencing symptoms [6]. While abnormal uterine bleeding and pelvic pain were this patient's main reason for surgical intervention, definitive surgical management also incorporates a preventative strategy for potential future sequelae related to subsequent cesarean scar defects if left uncorrected [1, 3].

Robotic-assisted laparoscopic management also enables precise fibrosis excision and subsequent layered myometrial repairs and reconstructions, potentially optimizing future uterine strength and minimizing future complications related to pregnancy or parturition [12–14]. While not formally examined in this case, this surgical management also incorporates a plan established in other studies demonstrating utility in optimizing existing residual myometrial tissue and existing uterine contour [6, 15].

These factors become essential in clinical decision making for osteopathic physicians involved in women’s health, minimally invasive gynecologic surgery, and perioperative care, as isthmocele represents not only a source of current symptoms but also a condition with meaningful implications for future gynecologic and obstetric health [1].

**Figure 2:**
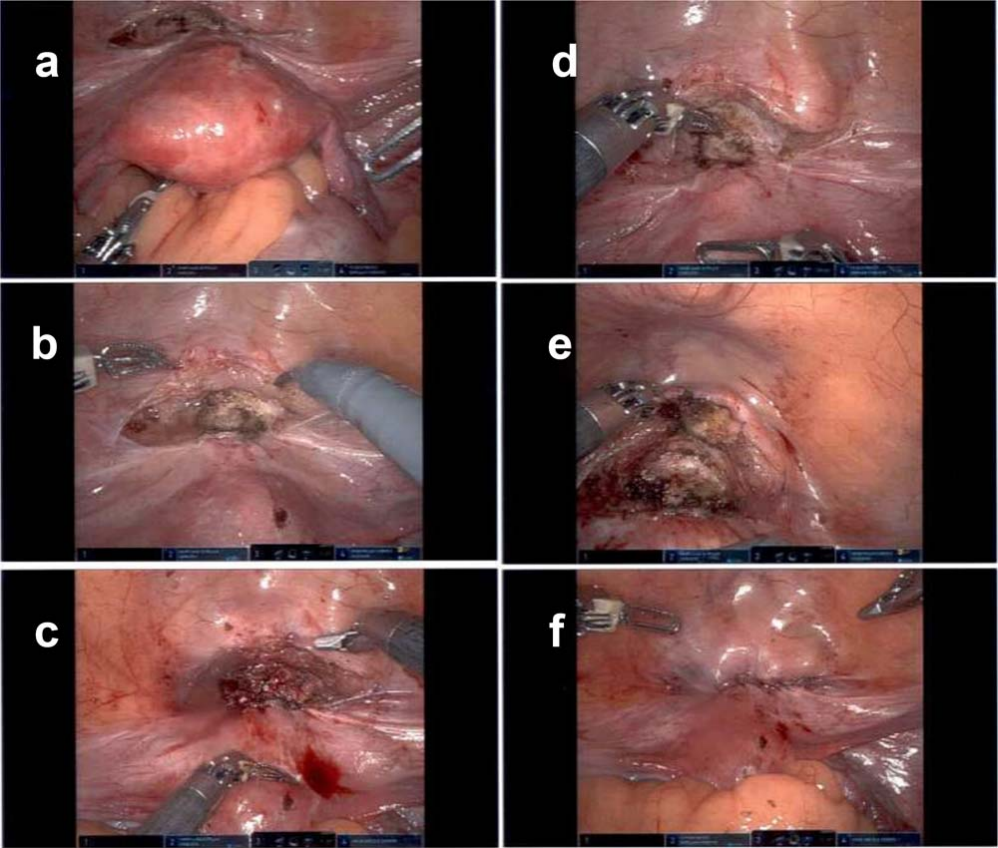
Laparoscopic intraoperative images demonstrating identification and repair of a uterine isthmocele defect; the figure depicts initial visualization of the isthmocele defect (a), closer inspection showing thinning of the myometrium (b), dissection and delineation of the defect margins (c), excision of fibrotic tissue within the defect (d), primary closure of the defect with suturing (e), and final appearance following complete repair (f).

Clinical outcomes following minimally invasive scar defect repair are favorable, with improvement in abnormal bleeding, pelvic pain, and fertility outcomes [7]. This case corresponds to such findings and provides intraoperative visualization (Fig. 2) that show defect morphology and the reconstructive technique.

## References

[1] Bij de Vaate AJ, van der Voet LF, Naji O et al. Prevalence, potential risk factors for development and symptoms related to the presence of uterine niches following cesarean section: systematic review. Ultrasound Obstet Gynecol 2014;43:372–82. 23996650 10.1002/uog.13199

[2] Wang CB, Chiu WW, Lee CY et al. Cesarean scar defect: correlation between cesarean section number, defect size, clinical symptoms and uterine position. Ultrasound Obstet Gynecol 2009;34:85–9. 19565535 10.1002/uog.6405

[3] Marotta ML, Donnez J, Squifflet J et al. Laparoscopic repair of post-cesarean section uterine scar defects diagnosed in nonpregnant women. J Minim Invasive Gynecol 2013;20:386–91. 23357466 10.1016/j.jmig.2012.12.006

[4] Osser OV, Jokubkiene L, Valentin L. High prevalence of defects in cesarean section scars at transvaginal ultrasound examination. Ultrasound Obstet Gynecol 2009;34:90–7. 19499514 10.1002/uog.6395

[5] Antila-Långsjö RM, Mäenpää JU, Huhtala HS et al. Cesarean scar defect: a prospective study on risk factors. Am J Obstet Gynecol 2018;219:458.e1–8. 10.1016/j.ajog.2018.09.004 30240650

[6] Roberge S, Demers S, Berghella V et al. Impact of single- vs double-layer closure on adverse outcomes and uterine scar defect: a systematic review and meta-analysis. Am J Obstet Gynecol 2014;211:453–60. 24912096 10.1016/j.ajog.2014.06.014

[7] Dominguez JA, Pacheco LA, Moratalla E et al. Diagnosis and management of isthmocele (cesarean scar defect): a SWOT analysis. Ultrasound Obstet Gynecol 2023;62:336–44. 36730180 10.1002/uog.26171

[8] Özüm G, Güraslan H, Deniz L et al. Isthmocele risk in repeated cesarean: the diagnostic and clinical role of morphometric parameters. Arch Gynecol Obstet 2025;312:2321–32. 41205040 10.1007/s00404-025-08238-6 PMC12705807

[9] Fabres C, Aviles G, De La Jara C et al. The cesarean delivery scar pouch: clinical implications and diagnostic correlation between transvaginal sonography and hysteroscopy. J Ultrasound Med 2003;22:695–702. 12862268 10.7863/jum.2003.22.7.695

[10] Vervoort A, van der Voet LF, Hehenkamp W et al. Hysteroscopic resection of a uterine caesarean scar defect (niche) in women with postmenstrual spotting: a randomised controlled trial. BJOG 2018;125:326–34. 28504857 10.1111/1471-0528.14733 PMC5811899

[11] Gkegkes ID, Psomiadou V, Minis E et al. Robot-assisted laparoscopic repair of cesarean scar defect: a systematic review of clinical evidence. J Robot Surg 2023;17:745–51. 36436106 10.1007/s11701-022-01502-w

[12] Surico D, Vigone A, Monateri C et al. Minimally invasive surgery for the excision and repair of cesarean scar defect: a scoping review of the literature. Medicina (Kaunas) 2025;61:1123. 40731753 10.3390/medicina61071123 PMC12299830

[13] Wang HF, Chen HH, Ting WH et al. Robotic or laparoscopic treatment of cesarean scar defects or cesarean scar pregnancies with uterine sound guidance. Taiwan J Obstet Gynecol 2021;60:821–6. 34507655 10.1016/j.tjog.2021.07.007

[14] Allameh Z, Rouholamin S, Rasti S et al. A transvaginal ultrasound-based diagnostic calculator for uterus post-cesarean scar defect. BMC Womens Health 2023;23:558. 37891612 10.1186/s12905-023-02715-3 PMC10612219

[15] Piróg M, Pulka A, Kacalska-Janssen O et al. Reproductive outcomes after surgical resection of isthmocele in secondary infertility. Int J Gynaecol Obstet 2025;169:746–51. 39665429 10.1002/ijgo.16080

